# Reply to Dr. Barry's Remarks on the Gibraltar Epidemic of 1828

**Published:** 1831-03

**Authors:** Hugh Fraser

**Affiliations:** late Surgeon of the Civil Hospital, Gibraltar.


### GIBRALTAR EPIDEMIC.

*Reply to* Dr. BARRY's *Remarks on the Gibraltar Epidemic of* 1828. By HUGH FRASER, Esq., late Surgeon of the Civil Hospital, Gibraltar.

> "As Cadiz, almost without quarantine regulations, remained free from epidemics from 1743 to 1799; and, with very rigorous quarantine regulations, suffered from eleven epidemics from 1800 to 1820, something must be behind the door, requiring examination."—Dr. SALVA, Professor of Medicine at Barcelona.

TO THE EDITOR.

SIR: You will merit thanks from the public for recording, so freely, details connected with yellow fever; for, although the subject be not of immediate interest to the mass of the professional men of England, it is one which, as to its contagious or non-contagious nature, "science, commerce, humanity, alike demand that the truth should be ascertained," as Dr. SOUTHWOOD SMITH has so well observed in his book on Fever.

To all who have followed the late discussion between Dr. BARRY and myself, on the subject of the Gibraltar epidemic

Reel: 81 - 36 - 4
(pp. 202-24, 304-18, 399-413)

of 1828, it must be quite evident that, were I only to consult my personal feelings, I perhaps could, after the publication of such a letter as his last, in your Journal for February, retire from the contest without discredit; for if, in former numbers of your Journal, I had been considered by that gentleman as a "cat's paw," as a disappointed man, "pushed on by a coterie," and as occupying myself with details, "not one word of which" belonged to me; I am now, by the pen of that very same gentleman, most thoroughly and satisfactorily purified. If, in common with many of my honourable colleagues of Gibraltar, I had been branded in a Journal as "crazy," as similar to the "pompous empty Musselman," and as something still worse than all this, I have, in common with my colleagues, been whitewashed and eulogised in your last Journal, by the same pen, to a degree sufficient to satisfy the most ambitious. Were it not, therefore, that now, as at all times throughout this discussion, I feel bound as an evidence in a *public cause*, I should, to consult my own taste, certainly prefer not appearing any more on the subject of the Gibraltar epidemic.

In admitting most readily that the style now adopted, and the ample avowals now made by Dr. Barry, should be met, on my part, by a wish to avoid hereafter any thing like asperity, or unnecessary severity of language, the cause of truth must not be one bit the less faithfully, less diligently, or less energetically advocated. If, with a view to enable the public to get at truth, I shew (which is quite unavoidable) how certain individuals have mystified things, I am not to be told, when I give their names, that I am influenced by personal motives, or that I am engaged in what Dr. Barry terms "personal polemics." I feel that this is an ungracious office; but truth, so much needed by the world at large on the subject of yellow fever, must be obtained even at this price. Since my last letter, the names of persons who have displayed bad faith in the affairs connected with the Gibraltar epidemic, have been given in publications in France, as well as in this country.

As, upon the origin of this epidemic, the evidence is conflicting, the public will, no doubt, in this, as in other cases, inquire a little into the good faith of those who present themselves as witnesses. Dr. Barry has himself removed the "*nefas*" which it once had pleased him to stamp on all the public witnesses who dared to give honest evidence against the cause of the superintendent of British quarantine; and now the public are enabled to judge how far certain witnesses, in favor of this last gentleman's cause, are now, by their own shewing, worthy of consideration, on a question of high importance. Dr. Barry doubtless merits credit for having so frankly unburdened his conscience, by doing justice, even at the eleventh hour, to those among whom, most assuredly, paltry ideas, foreign to the honour of their profession, were never harboured. I am willing to believe that, while he made such ample amends to the living, what was due by him towards the dead had escaped his memory: I allude to the late Dr. Hennen. To what Dr. Barry has said, in justice to the medical gentlemen of Gibraltar during the epidemic of 1828, it remains for me to add, (lest an impression should go forth as to the existence, among them, of a disposition to unbecoming "squabbling,") that, until the arrival of two gentlemen in the garrison, at the close of the epidemic, and whose names my readers can have no difficulty in tracing, never before, I believe, was there more cordiality, more perfect disinterestedness, or more liberality in giving mutual aid and advice. I fear that a portion of the public has been disposed to look upon the present discussion as tainted by a party spirit. Now, in truth, all Gibraltar non-contagionists were, to a man, those who, led by the evidence of facts, supported their opinions *in direct opposition to their personal interests:* they could very soon perceive that the way to be in bad odour in quarters where the power of doing an injury or conferring favors lay, was by declining to lend themselves to the side of contagion.* I call upon the public to bear in mind that, in all the Gibraltar epidemics, those medical men, professing themselves to be contagionists, have been, with very few exceptions, either persons holding profitable situations in the quarantine department, or those who hold those situations *in partibus;* or, again, those who have received promises, protection, or favors from persons holding the profitable situations alluded to. This should never be lost sight of in balancing the evidence on the question of the contagious or non-contagious nature of the black-vomit fever of Gibraltar. Not a word which can be considered as savouring of hostility or rancour shall be directed by me towards individuals supporting, honestly, opinions as to the contagious property of that fever; opinions founded on circumstances not duly examined, and which

* Indeed, all the ways in which the personal interests of Gibraltar non-contagionists were placed in jeopardy cannot be stated: never were circumstances so calculated to demoralize the profession. "Sir, you are not a safe person to remain in this garrison," said the superintendent of British quarantine, to Staff-surgeon Dix, in May 1829. "Fraser, it will be better for you to be on our side," said the superintendent's secretary (Dr. Barry) to me, in an interview which he pressed upon me, a few weeks previous.

they would probably not hesitate to confess were erroneous, on further investigation; as a great leader of Spanish contagionists (Dr. Piguellem) did, when he witnessed the epidemic of 1821 at Barcelona, as the author of the article "Fever," in the "Dictionnaire des Sciences Medicales" did, when he observed the course of the disease more closely in the West Indies; as the celebrated Rush did in America; and, in short, as many others have done.

I believe that, by the publication of Dr. Barry's paper, read at the College of Physicians, and his other published papers, (Code Sanitaire, &c.) the public must be now pretty nearly in possession of whatever that gentleman can furnish towards the support of the nearly prostrate cause, which has so long been a source of comfort, patronage, and emolument to certain persons; and it will be seen how little any degree of dexterity is able to triumph over truth.

The first paragraph deserving notice will be found by turning to No. 381, page 381, of your Journal,* namely, "Mr. Fraser tells you and your readers, that, before the arrival of Dr. Pym in the garrison, in November, even the word contagion had become nearly obsolete," &c. To disprove this, Dr. Barry has adduced the copy of a letter relative to hospital bedding, written to the military secretary, by the late Dr. Hennen, principal medical officer in Gibraltar, at the breaking out of the fever in question. It seems to me quite pitiable to attempt to prove that, because from that letter (penned so early in the epidemic season as the 24th of September,) it would appear that Dr. Hennen entertained fears as to the effect of a prodigious collection of foul hospital bedding, the word contagion had not become obsolete in Gibraltar after the lapse of a period of more than two months.

In the paper inserted in the review mentioned in the last note, I gave copies of letters from Dr. Hennen, shewing his entire conviction of the disease being of *local* origin; and, from having been in constant communication with him, I know he had the same conviction up to the hour of his death. Little could this talented man have imagined, when he wrote those letters, dictated by honest feelings of duty, that, after his death, they would be commented upon in a manner which I shall now forbear to characterize. Let the first paragraph of page 384 be looked at, and the insinuation there, so

* The pages quoted throughout this paper will always bear reference to Dr. Barry's rejoinder (published in No. 381, vol. lxiv. of this Journal) to my paper inserted in the Medico-Chirurgical Review, No. 26, fasc. 2, p. 337, unless the contrary be noted.

openly conveyed, will shew to what extremes the advocates of the advantageous side (the quarantine side) of this question will go to support what, I think, must be considered as untenable. Although Dr. Hennen was so decided in what he has stated in the letter alluded to, against the *foreign* origin of the fever, he was naturally justified, particularly with the high responsibility which rested upon him, in having some doubts as to the possibility of the disease becoming contagious from numbers being crowded together, and of the possibility also of contagion by *fomites*, in foul accumulated bedding. This, it is, I think, very reasonable to suppose had caused him, as a precautionary measure, to express himself in the manner he has done in the letter in question; but that, after sufficient observation, he maintained an opinion of this kind, does not at all appear to have been the case; nor do I think that such can be shewn by reference to any other of his writings, dictated during this lamentable period. Indeed, as to contagion there was not, I believe, at the beginning of the epidemic, a single medical officer of the garrison who had not doubts and apprehensions upon the subject; while, at its close, when the whole evidence was before them, there was not a solitary individual, that I know of, among those who witnessed the course of the disease throughout, who had any doubts left upon the subject; and some of those gentlemen had quite as much experience of diseases as Dr. Hennen. Even on the question of importation, two of them had, at the breaking out of the disease, written to Dr. Hennen, calling his attention to rumours as to the importation of the malady by a certain ship. The letters of these gentlemen are in the medical office at Gibraltar, and they shew, beyond all doubt, the unbiassed manner in which the question was at that moment met. Dr. Barry, in his zealous search after documents calculated to aid, in ever so feeble a manner, the prostrate cause of yellow fever contagion, could scarcely have failed to have seen those letters, which, I presume, must have convinced him that truth alone was sought after by those who, after this period, became decided non-contagionists. For my part, I cannot imagine how it is possible that any two persons, calling themselves contagionists in regard to yellow or Bulam fever,* can, if they have been at Gibraltar, and are in possession of all the occurrences of the year 1828, ever converse together without smiling at the

* I am prepared to shew, if necessary, that a certain celebrated advocate of the importation of this Bulam adopted what is termed expurgation, or purification of the town of Gibraltar, after the epidemic of 1828, "to please the Spaniards merely:" not then, as it would appear, to destroy any latent principle of contagion!

gullibility of a portion of the public, as soothsayers were, we are told, wont to do, in reference to their peculiar calling.

At page 381, Dr. Barry observes, "When Mr. F. shall have redeemed his promise of 'more of this hereafter,' relative to a paper which he states 'I drew up,' shortly after my arrival in Gibraltar, to prove the non-contagious nature and local origin of this epidemic, I shall reply; not before. I dare him to the proof." Those who are well aware that such a document was drawn up by Dr. Barry almost immediately after his arrival in Gibraltar, must be astonished at his intrepidity in thus daring me to the proof. As my proof, I beg to offer them the following letter from the French physician, Dr. Chervin, one of the members of a commission sent by that government to Gibraltar in 1828, to inquire into the nature and origin of our epidemic: this letter will, I think, shew, not only that I have made no vague statement respecting that document; but it will shew further, and on the evidence of one whose veracity I do not suppose Dr. Barry will be inclined to question, that this advocate of what I consider a very bad cause has woven a web for himself, from which it will not be very easy for him to emerge. Here is a translation of Dr. Chervin's letter.

"*Paris, Rue Villidot; Nov.* 29, 1830.

"My dear Mr. Fraser:

"You ask me whether, on my arrival in Gibraltar in 1828, Dr. Barry did not pronounce in favor of the *local origin* and of the *now contagious* property of the yellow fever, which then reigned in that garrison. The following is what he stated to me on two different occasions: I arrived at Gibraltar on the 23d November, and Mr. Amul (surgeon of the 12th Regiment) told me, on the 24th, at the Naval Hospital, that Dr. Barry, who lived in camp on the Neutral Ground, wished, very much, to see me. On the following day I went to the camp on Neutral Ground, and I had scarcely arrived when that physician came up to me, conversed with me on the prevailing disease, and among other things made use of these remarkable words: "M. Chervin, all the facts of which I have been a witness here, confirm completely (*entièrement*) your opinion." Now my opinion was then, as it still is, that the yellow fever is neither contagious nor imported. Mons. Trousseau was present at this conversation, and he heard perfectly the words which I have just quoted.

"Eight or ten days afterwards, Mons. Barry came to see us (the French commission) at Bell lane, at our breakfast hour: he renewed the subject of yellow fever to me, and was on this occasion much more explicit than on the first; he told me that the epidemic which reigned in Gibraltar was owing to an atmosphere stagnant, vitiated, corrupted, &c., and that he had accordingly applied to the lieutenant governor, to appoint a commission for the purpose of examining into, and deciding upon, the causes of the disease. M. Trousseau was again present at this conversation, and Dr. Gilkrest,

Deputy Inspector of Hospitals, just entered as M. Barry ended his energetic exposition of what he then looked upon as the causes of the disease. It was, therefore, not without surprise, that I saw that physician face about (*faire volte face*) a little after the arrival of Dr. Pym at Gibraltar, displaying zeal the most ardent for the doctrine of the importation of the disease.

"But there is one circumstance, of which you cannot be ignorant, which is, that M. Barry did not rest satisfied with merely expressing his opinion verbally, but wrote, as is stated, a long memoir in proof of the local origin of the epidemic. Mr. Moore, Assistant Staff Surgeon, attached to the hospital of Gibraltar, informed me, that he himself had copied this memoir of Dr. Barry, and that that physician had supported very strongly the indigenous origin of the disease. On the 13th March, 1829, Major Middleton, of the 42d regiment, also assured me, at the house of Mr. Duguid, merchant, and in the presence of the latter, that he had read the manuscript in question, and that the author shewed himself quite opposed to the doctrine of importation. Dr. Broadfoot had, as that gentleman informed me, the memoir of M. Barry in his possession for several days, but his occupations did not permit him to read it; Miss Broadfoot, who amused herself in looking over the manuscript, told me that it consisted of about fifty or sixty pages. In short, Mr. Wm. Sweetland informed me at Gibraltar, that he had read the memoir alluded to, and during his residence in Paris in the month of August last, he was kind enough to let me see a paper, which he himself had drawn up, full of interest, relative to the late epidemic of Gibraltar, and in which he thus expresses himself in English relative to the manuscript of Dr. Barry:

" 'Dr. Barry, after the most mature consideration of the question, at least I am bound to think so, declared that he saw cause sufficient in the place (Gibraltar) for the appearance of the disease, pointed out remedies, and submitted the result of his deliberations, in a memoir to Dr. Broadfoot, his official chief, with a view to its being sent to his Majesty's government. This memoir I have since understood was suppressed, that it was not forwarded to England. I cannot be mistaken as to the doctrines contained in it, for Dr. Barry paid me the compliment of sending it to me; I read it with much attention, and thought it, notwithstanding a great deal of wild speculation, which it contained, to be the work of a man of considerable acuteness of observation.'

"I beg, my dear Mr. Frazer, that you will excuse the length of my reply. I thought it proper to enter into some details, in order that you might be well convinced, that Dr. Barry did not always profess the opinion which he supports now; at least this has been proved by what he, without having been asked, told me at two different times, and by his memoir, which three persons worthy of belief, assured me they had read.

"Your devoted servant and friend,

(Signed) "—— CHERVIN, D.M.P."

"To Mr. Hugh Fraser."

In addition to the respectable witnesses cited in the above letter, I would appeal to Colonel Chapman, civil secretary, and to Colonel Marshall, military secretary, of Gibraltar, whether the memoir in question was not sent to them by Dr. Barry, for their perusal. Dr. Barry may, however, by a little effort, recollect that he read some pages of this production to me, in his tent on the Neutral Ground, one day, when I paid him a visit, in company with Major Cruise of the 12th regiment. The Doctor, I am sure, cannot forget having asked me whether there was any thing objectionable in what he had read to me? that is, if my views of the localities, &c. of the rock, and my ideas of the local origin of the fever, coincided with his? he must indeed remember, that I then fully concurred in what I had heard him read over. Dr. Chervin may well say, that it is not without surprise he witnessed the quick *volte face* of Dr. Barry, on the arrival of the medical chief of the British quarantine:* it afforded a frequent theme of conversation and amusement at Gibraltar; and, if I do not mistake, during the sittings of a commission, a little raillery was now and then employed on the subject by Colonel Chapman, one of the members of that commission.

In the above, I think, most people will find ample proofs that Dr. Barry was, previous to coming into contact with Dr. Pym, a believer in the domestic origin of our epidemic. From the manner in which I have been dared to prove that Dr. B. had so written, something in the shape of counter-proofs will, no doubt, be produced: if certificates from any individuals should be brought forward, it will not be requiring too much, I presume, that they should bear a date corresponding to that on which Dr. Barry's document left their possession. As it appears from the foregoing letter from Dr. Chervin, (and there are other proofs,) that Dr. Broadfoot, of the Gibraltar quarantine, did not read the document in question, although it had been sent to him for that purpose; a certificate, were it even to come from that gentleman, could not, I apprehend, prove the identity of a paper of many pages; unless, indeed, it be shewn that Dr. Chervin and others are wrong, and that Dr. Broadfoot did read the paper, and attached his certificate to it before it left his possession.

Happily for the cause of science, (happily, inasmuch as it explains the secret of Dr. Barry's uncommon zeal in support of the side of contagion,) he did not take up a ground of defence on the point in question, on the score of further

* This gentleman, as is now understood, solicited permission to go to Gibraltar at the close of the year 1828.

experience having led him to change his opinion; as many, unacquainted with the facts, would naturally have considered this a valid-enough reason, although, at Gibraltar, it was well known that the period between his handing about his paper on local origin, as well as of his *viva voce* declarations to Dr. Chervin, and that of his making *volte face*, was infinitely too short to produce such a change of opinion, had the Doctor been ever so well disposed to obtain correct information; but which, I think, we shall see, as we proceed, he was not.

Taking Dr. Barry's definition of yellow fever as we now find it in his paper read to the College of Physicians, and inserted in the last number of this Journal, page 94, namely, "This disease, reduced to its most elementary form of expression, consisted of one, and only one, febrile paroxysm, terminating, from the second to the sixth day, either in a rapid and permanent return to health, or in the precursory, but apyrectic signs of speedy dissolution," &c. This definition will not, I apprehend, be found very clear by any body. Does Dr. Barry wish people to understand that his "three acts of a drama," (p. 382, Rejoinder,) "the single paroxysm," lasted "from the second to the sixth day?" Whatever he wishes us to infer, it is clear, from what he has yet furnished the profession with, (in his Code Sanitaire,* as well as elsewhere,) that he subjects himself to the just criticism of his colleagues for his contumacy in writing on a disease, the nature of which he seems to know so little of; about which he could not possibly, in fact, know much, he having only, as I have elsewhere stated, arrived at the close of our epidemic: "*Medicina non ingenii humani partus est, sed experientiæ filia:*" but he should, at least, have taken the precaution to obtain correct information about it from others, before he ventured to draw up a paper for a learned body. Can it be necessary to enter at any length on his remarks on my statement, as to the yellow fever sometimes, in favorable cases, being of a continued form at its commencement, and observing remissions at its close? What is there at all strange in this? who has ever seen any thing of the fevers of warm climates, and not observed this? Dr. Barry's "perfect angles" and "circular forms" will not avail him in the crowded wards of a yellow fever hospital: better for him to treasure up what Sir John Pringle says relative to the varied types of fever. Is it necessary to point out how expressly Cullen notices the changes of type in fevers? Dr. Barry refers us to this author for a definition of fever: strange, however, the Doctor does not tell us

* Vide No. 382, p. 475, of this Journal.

that this celebrated nosologist declares, in the most pointed language, that, "in a practice of half a century," he had never witnessed a *six-day fever;* nor do I believe any other practitioner has, save the members of the superintendent of quarantine's school. I would recommend Dr. Barry to consult what that excellent practical physician, Dr. Elliotson, of St. Thomas's hospital, has lately said as to the insidious forms of remittent fever; namely, "Remittent fever will frequently occur insidiously, and, unless you are quite up to it, you may as easily pass it over as some forms of epilepsy. I have had many cases of remittent fever, which, in addition to the symptoms of continued fever, were merely characterized by excessive sweating." (London Medical Gazette, January 3d, 1831.) From the same source, I think, I may be allowed, *en passant*, to remark, that Dr. Barry may receive another excellent practical hint, namely, "It is wrong to suppose that malaria does nothing more than produce these particular forms of intermittent disease, [Dr. E. might well have added, and remittent forms also,] it poisons the whole body, and many persons are destroyed by it who never had ague at all, so deadly is the poison."

In a disease which, like yellow fever, presents, in different places, and in different epidemics, or even in the same place and during the same epidemics, so many varieties, grades, and anomalies; so many absolute *contradictions* indeed, as has been well observed by Spanish writers, in a disease which, out of their cabinets, it would puzzle the best nosologists to class satisfactorily; in such a disease, surely, every person is permitted to describe it as it has passed under his own eye. The passage quoted by Dr. Barry, from Mr. P. Wilson's paper, cannot be held as establishing that remissions did not, or do not, occur in this disease. Dr. Barry has stated, that the same patients were attended by Mr. Wilson and me: this was not the case; for, although our wards were in the same building, he (Mr. Wilson) had, during the epidemic, chiefly the charge of Spanish and other foreigners, while my patients were chiefly British; and our respective duties seldom admitted even of our communicating with each other. This fact, of the Bulam sometimes assuming a remittent form, the superintendent of quarantine has shewn himself most particularly tender upon, in his book printed in 1815;* as he conceives

* A book long since proved, by some of the most learned members of the profession, to be more full of errors, erroneous doctrines, and dogmatical assertions, than perhaps any other which has been presented to the world for a century. For a minute history of this curious work, see a "Sequel to an Essay on Yellow Fever," by Dr. Bancroft, and "Burnet's Mediterranean Fevers."

that establishing any connexion between the black-vomit fever and remittents must favor the doctrine of local origin; and hence, I believe, it is that we find Dr. Barry tender upon it also. But, besides the evidence of numerous British, French, and American writers as to remissions in yellow fever, and not to repeat that, quoted in my paper, inserted in the Medico-Chirurgical Review, from the celebrated Spanish author Arejula, (a contagionist, by the way,) Dr. Barry and the superintendent must permit me to refer, for the most irresistible evidence respecting this point, to some observations of Dr. Chervin,* attached to his translation of Mr. P. Wilson's paper on the Gibraltar Epidemic of 1828. What is there stated must place the matter quite out of the reach of cavillers. But what is, perhaps, more singular than all, is, that Dr. Barry should seem to be ignorant of the opinion, on this point, of one of the most eminent authors on yellow fever: "Never has the unity of our autumnal fever been more clearly demonstrated than in our present epidemic. Its principal grades, viz. the intermittent, the mild remittent, the inflammatory bilious fever, and the malignant yellow fever, have all ran into each other, in many instances. A tertian has ended in death with black vomiting, and a fever with the face and eyes suffused with blood, has ended in a quotidian which has yielded to a few doses of bark." (*Extract of a Letter from* Dr. Rush *to* Dr. Millar; New York Med. Repositary, vol. vi. p. 249.)

On other points, besides this, would Dr. Barry mislead all the colleges in Europe; the more particularly, as he proceeds step by step, and with no small tact, in support of the numberless errors, and false doctrines, which I have already stated are to be found in his patron's book; and which have all, long since, as I have stated in a preceding note, been most clearly refuted.

Regarding the insinuation, p. 383, against Dr. Hennen's conduct, as connected with his temporary duties in the quarantine department at Gibraltar, it needs only to be stated by me, that his correspondence proves that, far from seeking concealment relative to the reports connected with the ship Dygden, he particularly urged, in the early part of the epidemic, that a board, or commission, might be formed, to investigate all the circumstances connected with the origin of the

* This gentleman has been occupied, during sixteen years, in making investigations connected with yellow fever: he has, for the purpose of obtaining facts, visited nearly every part of the world, where the disease is known to have prevailed, and has published many most interesting pamphlets on the disease. —H. F.

disease; but motives were adduced for deferring the inquiry which he had solicited. Before Dr. Barry had scoffed so contemptuously at the name, however inappropriate, given to the disease by Dr. Hennen, it would have been well if he had ascertained that it had not been so named by such an authority, on yellow fever, as the celebrated Dr. Rush. As to the want of cleanliness, &c. (p. 384,) of some parts of Gibraltar, as reported upon officially by Dr. Hennen,* and attempted to be confuted by Dr. Barry, by an extract from Dr. O'HALLORAN's work on the Yellow Fever of Spain, it must be recollected that Dr. Hennen spoke of the town of Gibraltar as he inspected it in September 1828, and not of the town, generally, as it might have appeared to Dr. O'Halloran in 1823. Why not, on this point, have quoted the opinion of Dr. Broadfoot, (who succeeded Dr. Hennen as principal medical officer, and who was at one time, though not now probably, a contagionist,) as recorded on the proceedings of a board of commissioners, and in which opinion there are several paragraphs bearing on *local* causes of disease? No, no; a quotation, of this kind, could not have advanced the side of the question which Dr. Barry is pleased to advocate.

Referring to Dr. Barry's note (p. 383,) respecting information from Valencia, as to the ship Dygden having yellow fever on board; it seems strange that a report of this kind should come from the Mediterranean coast, respecting a vessel which had not entered that sea: the fact however is, a report of the kind did reach Gibraltar from the eastern coast, from *Alicant*, not Valencia; and the history of that report is, as the circumstances had been freely related afterwards at Gibraltar, that soon after the arrival of the ship Dygden at the latter port, a mercantile jealousy arose, from the consignment of the ship to one party instead of another, and, out of this, reports unfavorable as to the healthy state of the vessel were circulated. These reports found their way along the coast to Alicant; from whence they were echoed back, by the authorities of that place, to Gibraltar. No summer passes without reports of an alarming nature being circulated along the coast, from Cadiz to Barcelona; the subject of the occasional fearful visitor is eternally uppermost in people's minds, during the season favorable for its appearance; and it was but a very short time before the breaking out of our epidemic, that a most formal statement, of an alarming nature, had been sent

* On this subject, see, to some effect, the reports drawn up by a special commission, and given in a paper from Dr. SMITH, 23d regiment, Edinburgh Med. and Surg. Journal, No. 106, (1830.)

from Cadiz to that garrison, by a person in authority. The Spanish consul at Gibraltar could have had no proper authority for stating that "a bad fever raged at the Havannah," when the ship Dygden lay in that port, as may be seen by the copy of that ship's bill of health, given in Dr. Smith's paper, already quoted.

Referring next (p. 384) to the opinions of the members of a commission appointed to inquire into the origin of the epidemic, it must surprise every body, that not one of those who voted for importation, not even Dr. Pym, was bold enough to name the ship Dygden, or any other particular vessel, when they gave in their official opinions, in writing, at their last sitting: they merely dealt in vague general assertions, talked of presumptive evidence, and so forth. I cannot hesitate admitting that, in referring to the majority against importation, in my first paper, I committed a manifest error, in regard to numbers: as to moral weight of opinion, there was no error at all, as I shall be able, I think, to shew; and this was what I meant to express. When my paper was printed in the Med.-Chirurgical Review, I was, as I stated on a former occasion, at Gibraltar; and when it came into my hands afterwards, it was impossible to avoid seeing, that, when I had spoken of the opinions of the members of that commission, the omission of the words "of those uninterested," after "the majority at least," was a matter requiring notice, by myself, on the first opportunity. In point of fact, all medical men at Gibraltar, who had, from conscientious motives, resisted giving in their adhesion to "the cause" of certain persons of influence, were in the habit of denying any share of importance to the opinions of those who, as Colonel Chapman so clearly and forcibly expresses it in his opinion, "those who wish to prove it," (importation:) those medical men were, accordingly, in the habit of considering the point so far settled, that the majority of opinions of any value to the public was decidedly unfavorable to the idea of the disease having been imported.

As it may be important to shew how the commission in question was formed, I may state that the late colonial secretary (General Sir George Murray,) directed the formation of it. The lieutenant governor of Gibraltar (General Sir George Don) was appointed president; but his overwhelming duties, I believe, obliged him to delegate that office to Dr. Pym, a gentleman for some years past at the head of the medical department of the British quarantine, and who has been, more or less, connected with the Gibraltar epidemics, and with the local interests of the place, since the year 1804. Here is Dr. Pym's official opinion, given in, as previously and collectively agreed upon, at the last sitting: "From the very strong evidence before the board, of the first persons attacked by the late epidemic fever having been connected with the shipping, I am of opinion that the disease was of foreign origin; and that neither local nor atmospheric causes have had any share in its production." He adds about six more lines, in proof of the usually healthy state of Gibraltar. Throughout the superintendent does not name, for the benefit of the public, the ship by which the disease was imported: nothing, indeed, can be more vague.*

Besides Dr. Pym, the following were the members appointed, by order, from the colonial office: namely, Mr. Judge Howell, against importation: his explicit opinion is given in my first paper, and may also be seen in that of Dr. Smith, which has since appeared. Lieutenant-Colonel Chapman, of the Royal Engineers, civil secretary, against importation: his remarkable opinion, to be seen also in Dr. Smith's paper, as well as in mine.† Lieutenant-Colonel Falla, town major of Gibraltar, and at the head of the police department of the garrison, when the epidemic broke out: on him, consequently, devolved the responsibility of the cleanliness of the town, state of the drains, population, &c.: opinion against local origin, in consequence, as he has been pleased to say, of the "strong presumptive evidence," *ergo*, for importation. Mr. Sweetland, captain of the port: opinion against importation, but (from an over-delicacy, lest he should be considered as deciding in his own cause,) by inference only. This officer, as may be seen by looking at Dr. Smith's paper, allows others to draw an inference from no ship having arrived in the Gibraltar bay that season, (1828,) "with yellow fever on board," and from that disease not having "discovered itself among any of the shipping in the port." Dr. Broadfoot, medical officer to the Gibraltar quarantine department: although in possession of a

* The time has been, when such an opinion might have had some weight at Gibraltar, as well as in England; but the superintendent has found that, in the present day, this will not do. In Dr. Smith's paper (already quoted) we find that Dr. Pym did venture to name a ship as suspected, in one of his answers to some questions proposed to him by the Medico-Chirurgical Society of Cadiz. It is extremely well worth while to refer to Dr. Smith's paper, as it will be found he has exposed a variety of extraordinary mistatements and errors made by Dr. P. on this question.

† The straightforward and independent conduct of this officer, who very soon discovered how matters were wished, by some, to be conducted, will, for many years to come, be remembered in Gibraltar; and cannot be too much praised by all honourable men, lovers of truth, whatever may be their creed. "Those who wish to prove it" (importation,) "description of persons brought forward!" exclaims this soldier.

correct copy of this gentleman's opinion, as drawn up and officially delivered on the day agreed upon; its great length, though extremely worthy of being seen, precludes my giving it here. Dr. Broadfoot hazards an opinion for importation, though he acknowledges there are no "positive proofs." This gentleman feels that he is on slippery ground, for he says that "he cannot believe" the epidemic originated at Gibraltar; and "he cannot resist the persuasion," that if the ship Dygden "was not the sole cause, yet she was the principal means of bringing the late fever into the garrison!" Eminent logic for a desperate cause! But this is not all; for Dr. B. completely neutralizes all he says about importation, by a long string of paragraphs, pointing out various sources of local origin of fever; such as narrow and injudiciously built streets and dwellings, drains, population, &c. Dr. Broadfoot has often since (I have heard) been amused at the remarks passed on his opinion, which can be taken up on the most convenient side, like a vase with two handles. To the above members was added, by Dr. Pym's recommendation, Dr. Barry, then staff-surgeon; and through the same recommendation, I believe, was Dr. B. appointed secretary to the commission.* His opinion, as handed in on the closing of the proceedings, was merely as follows: "I am of opinion that the late epidemic was not of local origin, and was imported." Now, I go upon the authority of one of the members, when I state that, after all the evidence had been closed, and on a certain day, in the public room of the commission, it was formally agreed that, then and there, each member should state his opinion in writing, for the purpose of being forwarded, with the other proceedings of the board, &c., to England. This was accordingly done, and what each member stated was copied by some of themselves. Dr. Broadfoot wrote down a very long opinion, which he gave at the table of the board-

* It is, perhaps, worthy of notice, that, although this gentleman was not one of those named, by the late colonial secretary, to sit on the above commission, he had the "honour," as he has told the public, of being appointed a member of this commission; yes, and secretary besides. This statement of Dr. Barry, nobody, I believe, will be disposed to question; and I may, in truth, declare, that, in this double capacity, never was greater skill and energy displayed in any cause than was shown by Dr. B. on this occasion, in the cause of the superintendent of quarantine, who had recommended him to fill these offices. Is a secretary wanted for the commission of origin? Dr. Barry is named. Is a secretary wanted for the commission on the question of second attack of the fever, a question of the most vital importance to Dr. Pym? Dr. Barry is named. Is a person required to be near the gentlemen of the French commission, during their domiciliary visits at Gibraltar, for the purpose of procuring information relative to the fever? Dr. Barry is named!

room, from a memorandum he had previously prepared. When the proceedings had been thus closed, and the opinions were recorded, as agreed upon, in a collective body, no alteration, addition, or abstraction, could certainly be made, in regard to those opinions, with the least propriety, without the concurrence of the members. Dr. Barry will therefore, I trust, be able to exculpate himself from the imputation of bad faith in having extended his official opinion, which I take upon myself, from the incontrovertible authority of one of the members, to state was, as above, an affair of *sixteen words*, containing no mention of the ship Dygden, instead of a lengthened concern of *eighty-one* closely printed lines, as seen at page 397 of his Rejoinder. In this extended document, the Doctor advances various opinions and explanations, not only regarding the epidemic of 1828, but of former ones, and for the first time mentions the Dygden. But, take another specimen of the manner in which matters were managed by certain persons in Gibraltar at that time. At page 384, speaking of the opinions of some of the members of the board of commissioners, the gentleman, who had the honour of being secretary, says, "they (the opinions) are as follows." Now, they are not as "follows," in regard to that of Dr. Broadfoot; for, according to Dr. Barry, that officer's official opinion consists of a few lines only, while that which was officially given in by him at Gibraltar, for the purpose of being forwarded, by the secretary, to the colonial office in this country, consisted of no less than what now occupies, in a copy, *sixty-nine lines*, closely written on foolscap paper. What excuse is there, then, for a man who will thus add, and clip off, matters, wherever he finds it convenient for his side of the question to do so? Surely nothing can be more culpable, and the public will naturally draw their conclusions accordingly. The giving of Dr. Broadfoot's opinion entire was of great moment, as it indicated the embarrassment of the writer, who, although in the quarantine department, virtually admitted that he could not support that side of the question. The fact is, that the secretary possessed sufficient acumen to perceive that the part of this gentleman's opinion which he quoted, would, in the eyes of the public, be of little importance if the subsequent paragraphs of it appeared; accordingly, he suppresses them. It is certain that Dr. Barry does not say he is furnishing "extracts" of opinions: no, he observes, "they (the opinions) are as follows." Who, excepting those acquainted with the circumstances, on reading what is given of Dr. Broadfoot's opinion by Dr. Barry, will not believe that that is the whole of it. At page 385, Dr. Barry "hopes the

College of Physicians will induce our government to print, *pro bono*, the documents connected with this epidemic." By all means, let us have them; but, judging from the foregoing specimens, I apprehend that the printing of them would not be very agreeable to the gentleman who had the honour of being secretary.*

Among other offices which Dr. Barry had the honour of filling during the investigation at Gibraltar, was that of collecting information from persons who were afterwards to appear, or not, perhaps, just as the case might be, before the commission. From some circumstances which transpired respecting the evidence of a woman named Mary Parodi,† brought forward to prove the importation of the disease; also a man named Leaky,‡ employed in the scavenging department, brought forward to prove the innocuous nature of the drains; besides other persons. Colonel Chapman, who suspected all was not right, thought proper to move, that no *private* examination of the witnesses should take place: this proposition was, however, negatived by a majority of the board; the majority being Drs. Pym, Broadfoot, Barry, and

* I may state a simple fact with respect to myself; that an important letter, (as I considered it to be,) bearing date 17th April, 1829, which I addressed, through the secretary, to the commission, relative to the nature of black vomit fluid, and adverting to the case of a Jew, named Abraham Bensimon, whom I had strong proofs (which proofs I offered to submit to the commission, but which, I fancy, the secretary afterwards forgot to call for) of his having died of the Bulam fever, from his having, along with other symptoms, ejected quantities of black matter from his stomach previous to death, as early as the month of May, 1828, was suppressed by somebody; and which, after the proceedings had been sent to England, was called for by the colonial secretary.

† A woman described by Mr. Wilson, "quite notorious as being one of the most abandoned and dishonest characters in the garrison," (vide Dr. Chervin's translation into French of Mr. Wilson's paper, p. 23.) Dr. Pym had been cautioned against bringing her forward as an evidence of a proper kind, by one of his oldest friends in the garrison: nevertheless, Mr. Wilson discovered Dr. Barry receiving communications from this creature, in his tent in camp, previously to her appearing as an evidence. Perhaps, it may be now understood what Colonel Chapman meant by the observation attached to his opinion as to "the description of persons brought forward" to prove the importation of the disease.

‡ A rather curious circumstance arose out of this *employé's* evidence: he stated that twelve men had commenced the repairing, &c. of a certain line of drains during the epidemic; and that twelve men, when the work was ended, were found alive and well. No plainer proof could there be of the little influence possessed by the drains in the production of our fever; however, on a closer examination, by Mr. Judge Howell, who saw reason to be a little particular, the *ruse* here attempted was discovered: it came out that the twelve men found alive and well at the close of the job, were not the identical twelve who had commenced it; several of the original having actually sickened while at work, (six of them, I think,) and whose places had been supplied by others, so as to leave the number twelve always complete.

Colonel Falla. Colonel Chapman next moved, that all *private* information, of which a considerable quantity was understood to have been sent to the president and secretary, should be communicated to the commission collectively: this proposition was also negatived by the same majority; upon which, this independent member insisted that the witnesses, when examined before the commission, should (to use his own language,) "have their chair removed from under the *elbows* of the president and secretary," to the farther end of the table, so that they (the witnesses) might not be subjected to embarrassment from any of the members, in what they had to say to the commission.

We now come to the testimony of the woman Fani, whose husband and children, as had been said, introduced into the garrison the Bulam fever, from *some ship* in the bay. It is quite true that, under the system* of Dr. Barry, who was present, this woman did, on a certain day, give her evidence to the French commission, in an inconsistent manner; but it is equally true that, in the Doctor's absence, she afterwards gave her statement before a notary public, (a strong contagionist, and importationist, by the by,) in a most consistent manner, as I have lately shewn in the Medico-Chirurgical Review, No. 26, p. 343. A memorandum, however, undoubtedly appears in the collection of documents made at Gibraltar by the gentlemen of the French commission, who made domi-

* The following letter will illustrate what is here meant by the word system.

" *Gibraltar; 20th January*, 1829.

" My dear Sir,

" In reply to your letter of yesterday, I have to observe, that when I joined the commission of investigation, I expected that we were to proceed in the pursuit of facts, and not to search for circumstances with which to prop any preconceived or adopted opinions. In those expectations, however, I was soon disappointed: for I saw, daily, more and more, the disposition and actual attempts to torture facts to the contagious side of the question; and therefore, as I could not submit to the idea of putting my name to papers officially drawn up in this partial way, I thought it best to withdraw myself entirely.

" In candid inquiry I will go as far as any man, ability permitting; but to be led by one name, or another name, or for opinion, or for the prospect of gaining admission at preferment's gates: no, sir; to such I shall never prostitute my independence.

" I am, my dear sir, yours very truly,

(Signed) " P. WILSON."

This is a copy of a letter written to one of the gentlemen of the French commission (Dr. Chervin,) who, at the time, requested that Mr. Wilson would continue his services in assisting that commission to procure information. Dr. Chervin, in his preface to the French translation of Mr. Wilson's paper, in alluding to the above transactions, remarks, that Mr. Wilson's reproaches against Dr. Barry are " *malheuresement trop fondés*."

ciliary visits after the epidemic had ceased, purporting that Mrs. Fani misled or deceived them, ("*les trompait;*) but it appears that one of the gentlemen (Dr. Chervin) who signed it, did so under a firm belief that the words inserted were, that she herself had been mistaken or misled, (*se trompait;*) and, accordingly, when this document, with the others, was about to be printed lately in Paris, Dr. Chervin, previous to giving his authority for its passing through the printing office, demanded that a note, explanatory of his signature being attached to it, contrary to his conviction of what the real state of the case was, should be appended thereto. In this he was unsuccessful, after a correspondence upon the subject with his colleagues, Drs. Louis and Trouseau, who had, for very good reasons, sufficient influence with one of the ministers (Mons. Boisbertrand,) of the ex-king of France, in whose hands the quarantine department lay, to have the documents printed without its being accompanied by the usual voucher of special assent from Dr. Chervin to the printing office. All this has been certified, in a paper transmitted to me by Dr. Chervin, but which is by far too long for publication here. I am, however, happy to find that that gentleman is soon likely to give the public a full detail of this matter, together with details of many other curious events which have occurred between him and his colleagues of the French commission. But if Mrs. Fani had really shewn so "palpable a determination to mislead" the commission, as Dr. Barry says she had, why has the secretary not also also shewn that, when this woman was examined before the English commission, she, on that occasion likewise, attempted to mislead? No, her answers were given with the most perfect clearness and consistence. Here is what was elicited from her on that occasion:

Q. "Were Salvador and Catalina Fani your children, and do you know the cause of their illness and death, in August last?"—A. "They were my children: I do not know in what month they died; they died within five days of each other. I do not know the cause of their illness: the doctor said, they had a *tabardillo* and indigestion. They were attended by Dr. Lopez, who is dead: he said that the indigestion was the cause of the *tabardillo.*"

Q. "What employment did your husband and sons follow at the time of the death of your children?"—A. My husband was a cigar maker, but he did not go on board, either to buy tobacco, or to sell cigars."

Q. "Did your husband, or your two children who died, go into the bay at any time during the last summer or autumn?" —A. "No."

Q. "How do you know that they did not go into the bay?"—A. "Because, if they had, they would have told me; and they did not tell me."

The above answers are in perfect harmony with her declaration made before the notary public above alluded to, and, if more details were not given by this woman to the commission, it was because the tact of certain persons perceived that no good, to their cause, could arise from further questions. The secretary was, on occasions of this sort, admitted by all to display the most consummate skill.

Respecting the disease of which the two children of Mrs. Fani died, how very absurd it is to observe Dr. Barry bringing forward an entry in the register of the Roman Catholic church of Gibraltar, as proof of their having died of fever, when Dr. B. must know, or at least he ought to have acquainted himself with the circumstance, that the persons who make these entries take the disease from what any of the attendants, or next-door neighbours, choose to call it; unless, as it sometimes happens, a medical certificate may be forwarded to the vicar, from the attending physician; but which was not done in so far as the children in question were concerned. But, if further accuracy be claimed in this particular instance, why were those children not registered as having died of *yellow* fever, when we have Dr. Barry affirming, on loose authority, such as was very current at Gibraltar, that they were observed to have been yellow when put into their coffins; and that they had had black vomit, as stated by the noted *Mary Parodi*. As to the certificate of *John Cortez, surgeon,** I shall, besides the mother's affidavit, made before the notary public, already alluded to, and her examination before the British commission, just quoted, oppose to it also that of an Italian physician (Dr. Tassorilli,) present during the epidemic, to whom Dr. Lopez, the gentleman who attended them, had stated respecting their disease, precisely what it appears he had stated to the mother of the children, viz. that they had died from a *tabardillo*, occasioned by an indigestion; but not

* This is the "esteemed" and "vigilant friend" mentioned in Dr. Barry's Code Sanitaire, published in No. 382 of this Journal. I should regret if I said any thing here that could hurt the feelings of Mr. Cortez, whom I know and believe to be a very good kind of man; but it is right the public should know, on the authority of the late Staff-surgeon Glasse to Dr. Burnett, that Mr. Cortez practises physic within the boundaries of Gibraltar solely under a licence from the British superintendent of quarantine. If the history of former Gibraltar epidemics be referred to, it will be seen that "my esteemed and vigilant friend, John Cortez," is frequently mentioned. During our last epidemic there was only one person who outstripped him in zeal for his patron's cause.—H. F.

one syllable about *yellowness of skin*, or vomiting of *black matter*.

In point of fact, the ascertaining the nature of the diseases of which these children died, is of little, if any, importance to the cause of truth, as far as the question of importation is concerned; for, in the first place, notwithstanding the efforts of Colonel Chapman's importation friends, "those who wish to prove it," there was so complete a failure in establishing the affirmative on that side, that not one of the four members of the commission, who gave their official opinions in support of the disease having been introduced from a ship, ventured openly* to name a ship. In the second place, a disease could not have been brought on shore, from the bay, by individuals who had never been traced to have been on board any ship in that bay; and the more particularly, when it was shewn, beyond all cavil, not to have existed on board either the ship Dygden, or any other vessel in the bay.† Let it be observed, that the health-guard, Testa, who had been for many days on board the Dygden, was not taken ill until the *malaria*, or whatever people are pleased to call it, had affected the inhabitants generally of the district, No. 24, in which he resided, and long after he had been on shore: besides, his sister, who had not been on board, was taken ill before him. Thirdly, the actual nature of the children's disease is rendered of comparatively little importance, as it is beyond the possibility of doubt, from various sources of evidence already before the public, that not only did cases of genuine Bulam, or true unequivocal yellow fever, occur about the same time of their

* We have already seen how the worthy secretary has tried to patch up this deficiency by afterwards adding, (in a document purporting to contain his opinion, and unknown to the commission collectively,) the name of a ship, with other weighty matters. We also know, by Dr. Smith's paper, that it was only in certain answers to questions, put by the Medico-chirurgical Academy of Cadiz to the British superintendent of quarantine, that he (the superintendent) ventured to name the ship Dygden, as being, as he has there been pleased to say, "justly suspected." Indeed, Dr. Chervin doubts, and I think with very great propriety, that those answers to the Cadiz Academy were at all furnished by Dr. Pym; for it appears, that he assured Mr. Peter Wilson, both in Gibraltar, and in London, "of his thorough disbelief of the Dygden having been the medium of the fever's introduction." He also told Mr. Sweetland, late captain of the port of Gibraltar, and one of the members of the British commission, the same thing. Dr. Chervin, who notices these circumstances in his translation of Mr. Wilson's paper, with notes, to be had, I believe at the French medical booksellers in London, says at page 27, "Dr. Pym also told me several times, during my residence in Gibraltar, what he repeated to me in August 1829, when I had the honour of seeing him in Paris, that he did not think it was the Dygden that introduced the yellow fever into the town of Gibraltar."

† Vide Mr. Sweetland's official opinion, given in Dr. Smith's paper.

having been taken ill, but long before that period. Proofs of this are to be seen in Mr. Wilson's valuable paper, in Dr. Smith's very excellent observations on Dr. Pym's answers, printed at Cadiz, and published in the last number of the Edinburgh Med. and Surg. Journal; and we have also the evidence of the late talented Dr. Hennen, as quoted by Dr. Smith, to this effect, and corroborated by Assistant-surgeon Woods, of the 94th regiment. Mr. Woods has freely stated to me, and to various other persons, that Dr. Hennen, on a certain day, about the end of July, 1828, told him that he had just seen a case of the purest form of yellow fever, and which had terminated fatally; but as it will be necessary, a little farther on, to advert, in a more particular and detailed manner, to the existence of such cases, I shall say nothing more respecting them here.

On this part of our subject, it only remains for me to notice the following question from Dr. Barry: "Which," says he, "do you think would be most likely to deal in complicated made-up falsehoods, a boy not quite thirteen years of age, and a few washerwomen, or a hardened veteran smuggler?" designating by this epithet Mrs. Fani, whose husband and children were stated by a boy, named Coffiero, to have been on board *some ship* in the Gibraltar bay. Now, it appears that this innocent youth was the special protegé of the celebrated Mary Parodi, already mentioned, and, in fact, actually lived in her tent for about three months, in that same camp (Neutral Ground,) in which Dr. Barry was known to have had private communications with her, on the subject of the importation of the fever; so that the accounts of the *two ways* of importation given by the secretary, seem, after all, to have had one and the same source, and that not at all a creditable one. This noted woman Parodi, let it be observed *en passant*, although her services occupy a very prominent place among the memorabilia of importation, has not been once named, either by Dr. Pym, in his official opinion as president of the British commission, nor in his answers to the Cadiz Academy. As to the epithet of a "hardened veteran smuggler," given by Dr. Barry to the woman Fani, with a view, no doubt, to throw discredit on her evidence, as well as to give a colouring to the probability of her family having been employed in contraband tobacco trade, it is only necessary to say that Gibraltar is a free port, and that people may bring tobacco on shore there in a boat, as stated, with the same freedom as they may potatoes: nevertheless, whatever old Fani may have been capable of doing in his younger days, all parties shew him to have been utterly incapable, from age and infirmities, of following the dangerous and laborious pursuits of a Gibraltar smuggler* long previous to his death; or even of his capability, or the probability, of his having been sailing about the bay for pleasure, during the ship Dygden's quarantine; and if his honest relict interfered in such a traffic, it is, perhaps, the first instance of a woman, in Gibraltar, engaging in such a vocation. But it has been clearly shewn that old Fani had an attack of the epidemic fever in 1804: if, therefore, Dr. Barry shall continue to persist in saying that the old man died from an attack of the epidemic of 1828, the Doctor will allow the case, I presume, to have been a *second* attack of Dr. Pym's Bulam. He will thereby dislodge himself from his own, and from his patron's ground.

(To be continued.) p. 304,

---

### HOSPITAL REPORTS.

* Persons who export tobacco from the merchants of the Rock to the Spanish coast.

