# Erratum in the Last Number

**Published:** 1831-04

**Authors:** 


Erratum *in the last Number.*

P. 218, 14 lines from top, for Leaky, read Beaty.

